# Loss of ZBRK1 Contributes to the Increase of KAP1 and Promotes KAP1-Mediated Metastasis and Invasion in Cervical Cancer

**DOI:** 10.1371/journal.pone.0073033

**Published:** 2013-08-22

**Authors:** Li-Fang Lin, Chien-Feng Li, Wei-Jan Wang, Wen-Ming Yang, Dennis Ding-Hwa Wang, Wen-Chang Chang, Wen-Hwa Lee, Ju-Ming Wang

**Affiliations:** 1 Institute of Basic Medical Sciences, National Cheng Kung University, Tainan City, Taiwan, Republic of China; 2 Institute of Bioinformatics and Biosignal Transduction, National Cheng Kung University, Tainan City, Taiwan, Republic of China; 3 Molecular Inflammation Research Center, National Cheng Kung University, Tainan City, Taiwan, Republic of China; 4 Infectious Disease and Signaling Research Center, National Cheng Kung University, National Cheng Kung University, Tainan City, Taiwan, Republic of China; 5 Department of Pathology, Chi-Mei Medical Center, Tainan, Taiwan, Republic of China; 6 Institute of Molecular Biology, National Chung Hsing University, Taichung, Taiwan, Republic of China; 7 Graduate Institute of Medical Sciences, College of Medicine, Taipei Medical University, Taipei, Taiwan, Republic of China; 8 Department of Biological Chemistry, Union Council Irvine School of Medicine, California, United States of America; INRS, Canada

## Abstract

ZBRK1, a zinc finger protein that interacts with breast cancer 1 (BRCA1) and KRAB-ZFP-associated protein 1 (KAP1), has been suggested to serve as a tumor suppressor via repression of tumor metastasis/invasion. To date, the detailed molecular mechanisms for how BRCA1 and KAP1 participate in ZBRK1-mediated transcriptional repression, metastasis and invasion as well as the associated clinical relevance remain unclear. In this study, we demonstrated that both the N- and C-terminal domains of ZBRK1 are important for inhibiting cell proliferation and anchorage-independent growth in cervical cancer. Specifically, the N-terminal KRAB domain of ZBRK1 displayed a more crucial role in inhibiting metastasis and invasion through modulation of KAP1 function in a transcriptionally dependent manner. The loss of ZBRK1 results in an increase of KAP1 expression, which enhanced migration and invasion of cervical cancer cells both the *in vitro* and *in vivo*. Moreover, an inverse correlation of expression levels was observed between ZBRK1 and KAP1 following tumor progression from in situ carcinoma to invasive/metastatic cervical cancer specimens. Taken together, the current results indicate that a loss of ZBRK1 contributes to the increased expression of KAP1, potentiating its role to enhance metastasis and invasion.

## Introduction

The malignancy of cancer stems from its ability to invade and metastasize neighboring and distal tissues, resulting in local and systemic organ failures. Metastasis is a complex process that often requires tumor cells to break away from the cancerous tumor, invade through the extracellular matrix, and undergo intravasation into the bloodstream or lymphatic circulatory system, followed by extravasation and entrance to distant organs, allowing for the growth of micrometastases [1]. Many aspects of this complicated process are currently unknown, such as the gene expressions of the well-characterized metastatic suppressors.

More than 700 zinc finger proteins have been identified in humans. Zinc finger proteins bind to specific DNA sequences and switch genes on or off. Nearly one-third of mammalian zinc finger proteins have a highly conserved Kruppel-associated box (KRAB) motif. The functions of KRAB-zinc finger proteins differ significantly between species in regulating gene expressions, especially for those involved in transcriptional repression activities. Human ZBRK1 is a transcriptional repressor that contains an N-terminal KRAB domain, eight consecutive C2H2 zinc fingers, and a BRCA1-dependent C-terminal transcriptional repression domain (CTRD) [2]. ZBKR1 has the potential to regulate different downstream genes by interacting with a diverse group of proteins. For example, it was reported that ZBRK1 can inhibit the expression of the angiopoietin 1 gene via interaction with transcriptional corepressors, CtIP and BRCA1 [3]. In addition, KAP1 interacts with ZBRK1 through the N-terminal KRAB domain of ZBRK1. ZBRK1 and KAP1 also contribute to oriLyt replication efficiency through BBLF2/3 interaction [4]. To date, the detailed mechanisms and related cellular functions of the ZBRK1’s interactions with these proteins remain unexplored.

The primary amino acid sequence of KAP1 contains several conserved motifs: a RING finger, B-boxes, a coiled-coil region, a PHD finger and a bromo domain. The RING finger, B-boxes, and coiled-coil are collectively called the RBCC domain, which has been shown to be sufficient for homo-oligomerization and KRAB repression module binding [5,6]. Several studies indicated that KAP1 binds to KRAB-containing proteins and facilitates KRAB-containing protein-mediated transcriptional repression. Moreover, KAP1 also functions as a molecular scaffold to regulate chromatin structure, mediated through interaction with Mi-2a, a component of the NuRD histone deacetylase complex [7], methyltransferase SETDB1 [8] or heterochromatin protein 1 (HP1) family members [9,10]. Recently, KAP1 was reported to contribute to the inhibition of E2F1-mediated apoptosis [11] and correlates closely with poor prognosis in gastric cancer [12]. Additionally, KAP1 was described as playing a role in fibroblast-specific protein 1-mediated epithelial-mesenchymal transition [13]. Despite these recent efforts in elucidating its cellular functions and associated molecular mechanisms, the roles of KAP1 in tumorigenesis remain largely unknown.

Our previous study suggests that ZBRK1 acts as a metastatic suppressor and that the loss of ZBRK1 enhances MMP9 transcription in cervical cancer [14]. In the current study, we find that ectopic expression of KAP1 in HeLa cells significantly increases cell migration/invasion. This study provides the first direct evidence to demonstrate that KAP1 has the ability to promote tumor metastasis both *in vitro* and *in vivo*. In addition, ZBRK1 suppresses this process by recruiting BRCA1 through C-terminal interaction and inhibiting KAP1 transcriptional activity. Furthermore, in the cervical cancer specimens that we validated, the expression levels of ZBRK1 inversely correlated with the loss of KAP1. Collectively, these results suggest that ZBRK1 serves to inhibit tumor metastasis and invasion of cervical cancer through modulation of KAP1.

## Results

### KAP1 and BRCA1 interacted with the N- and C-terminal domains, respectively, of ZBRK1

**Figure 1:**
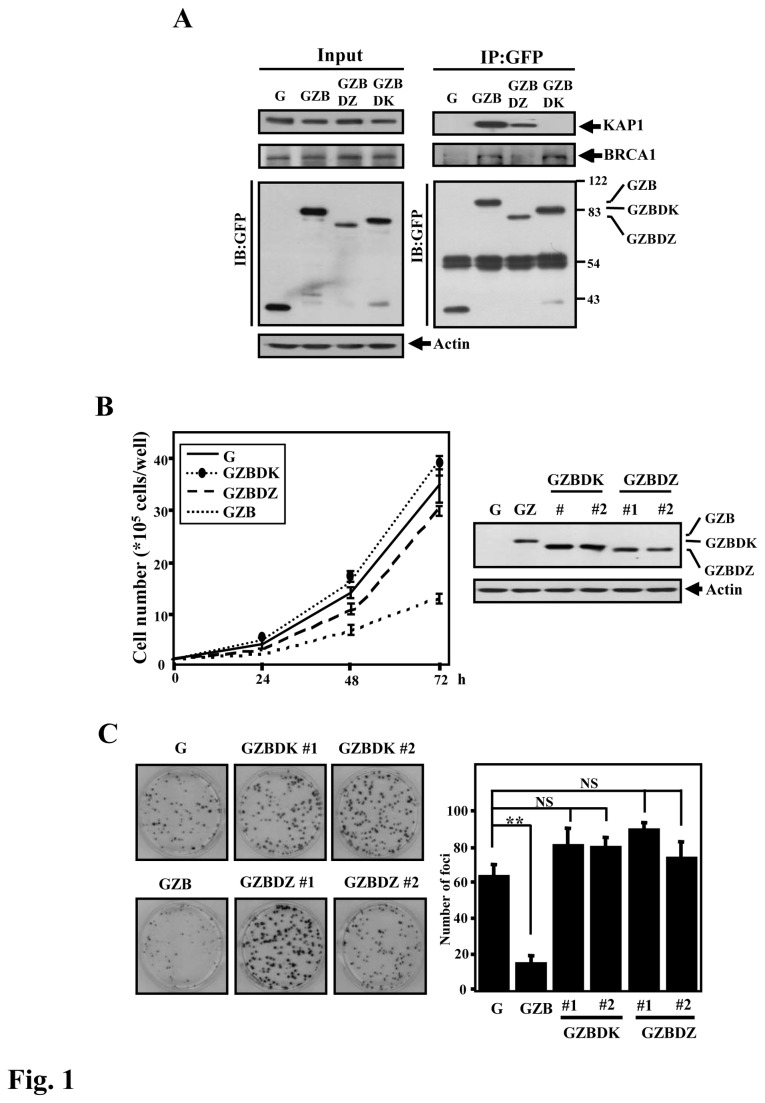
Both the N- and C-termini of ZBRK1 are required for ZBRK1-mediated inhibition of cell proliferation. **A**, The in vivo interaction between ZBRK1 and KAP1. The lysates of HeLa cells stably expressing EGFP (G), EGFP-ZBRK1 (GZB) or EGFP-fused truncated ZBRK1s (GZBDK and GZBDZ) were immunoprecipitated using an anti-GFP Ab (Roche). Immunoprecipitated proteins were eluted by first boiling in SDS sample buffer and then separating on a 10% SDS-PAGE gel, followed by immunoblot analysis with an anti-KAP1 antibody (Bethyl Laboratories) to detect KAP1 or with an anti-GFP Ab to detect GFP. **B**, Truncated N- or C-terminus of ZBRK1 loses the ability to inhibit growth. *Left*, equal numbers of stable HeLa cells expressing EGFP (G), EGFP-ZBRK1 (GZB) or EGFP-fused truncated ZBRK1s (GZBDK and GZBDZ) were seeded to assess growth by crystal violet staining. The viable cell number was determined at the indicated times. *Right*, the expression of ZBRK1 and its mutants in HeLa cells was analyzed by Western blot. **C**, Truncated N- or C-terminus of ZBRK1 loses the ability to inhibit proliferation. A focus formation assay was conducted using various stable HeLa cell lines. *Columns*, focus numbers; *bars*, mean ± SD. **p < 0.01 compared with control cells (G) using Student’s *t*-test; *NS*, no significance compared with control cells (G).

Previously, we found that ZBRK1 acts as a tumor suppressor, inhibiting the proliferation, migration, invasion and metastasis of cancer cells [14]. As mentioned above, KAP1 and BRCA1 interact with the N- and C-terminal domains, respectively [15,16], of ZBRK1. We were specifically interested in understanding the functional contributions of these interactions to tumor suppression. We first assessed the binding specificity of KAP1 and BRCA1 to wild-type or truncated ZBRK1 using an immunoprecipitation assay. The result showed that KAP1 and BRCA1 indeed specifically interacted with the N- and C-terminal domains, respectively, of ZBRK1 in HeLa cells (Figure **S1** and Figure **1A**).

### Both the N- and the C-Termini of ZBRK1 Suppressed Cancer Cell Proliferation, but Only the N-Terminus of ZBRK1 Inhibited Cell Migration

To address the cellular functions of ZBRK1, we created several stable HeLa cell lines ectopically expressing EGFP (G), EGFP-fused full-length ZBRK1 (GZB), or an EGFP-fused ZBRK1 deletion of the N-terminal KAP1-binding region (GZBDK) or the C-terminal BRCA1-binding region (GZBDZ) (Figure **1B** **, right panel**). An increase in ZBRK1 results in the inhibition of proliferation [14], so we first assessed the growth inhibition efficiency of these stable HeLa cell lines. Compared with GZB-expressing cells, both GZBDK- and GZBDZ-expressing cells lost growth inhibition. The result suggested that both KAP1 and BRCA1 interactions were necessary for ZBRK1-mediated growth inhibition (Figure **1B** **, left panel**). The same stable cell lines were used to further assess the effects of the KRAB and CTRD domains of ZBRK1 on cell proliferation using a focus formation assay. Similar to the observation in the right panel of Figure 1B, GZBDK and GZBDZ-expressing HeLa cells have no effect on cell proliferation compared with ZBRK1-expressing cell lines (Figure **1C**).

**Figure 2:**
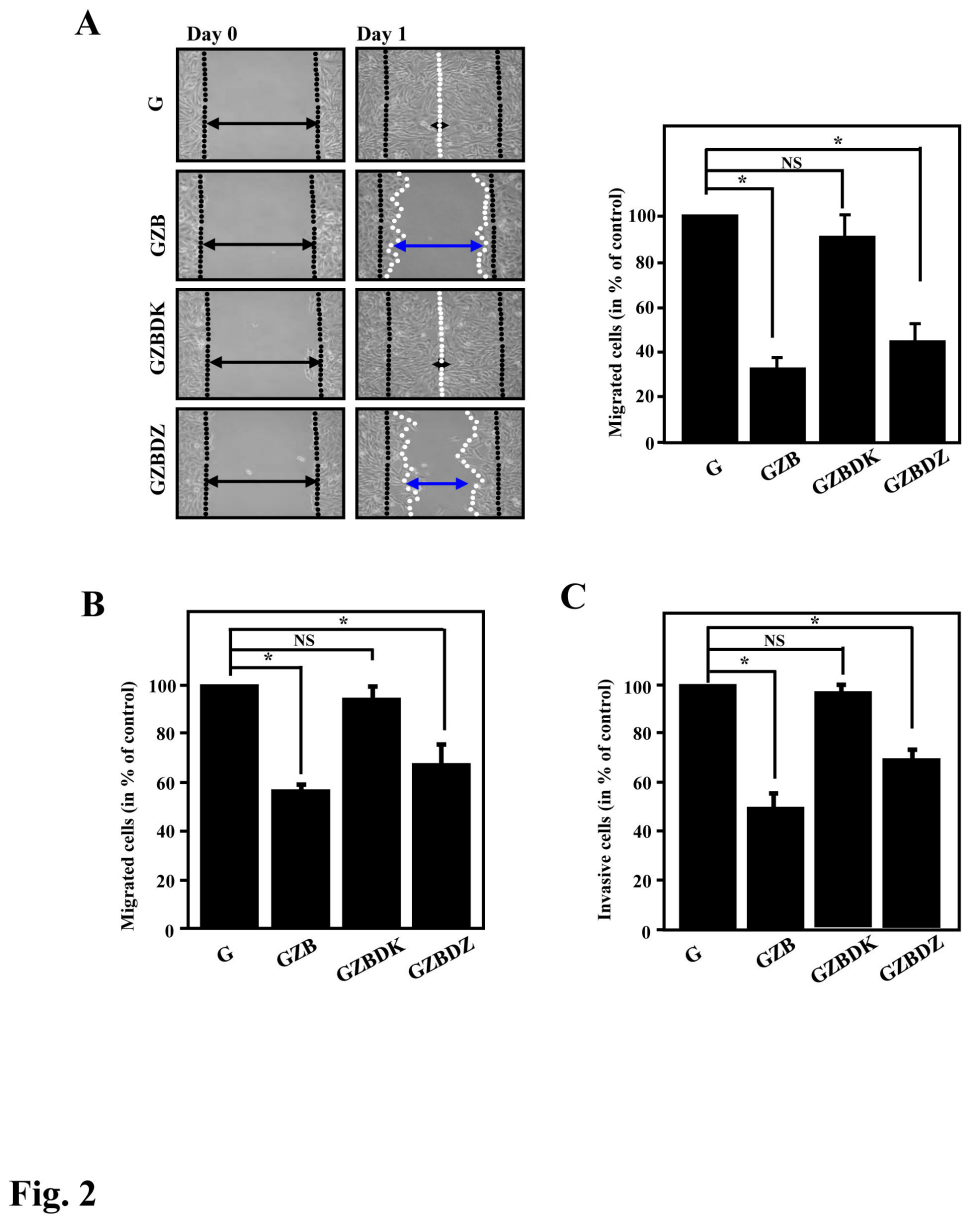
The KRAB domain of ZBRK1 is essential for the inhibition of cell migration and invasion. **A**, *Left*, a wound-healing migration assay was performed with HeLa cells expressing EGFP (G), EGFP-ZBRK1 (GZB) or mutated ZBRK1 (GZBDK or GZBDZ). Representative images of the wound sealing were collected on the day of the laceration and the day after the wound scratch. *Right*, the level of cell migration into the wound scratch was quantified as the percentage of wound healing. *Columns*, average of three independent measurements; *bars*, mean ± SD. *p<0.05. **B**, HeLa cells expressing GZBDK relieves the cell migration inhibitory ability. The cells were seeded in transwells, and the level of cell migration was determined. **C**, HeLa cells expressing GZBDK relieves the cell invasion inhibitory effect. The cells were seeded in a BD matrix gel layer, and the level of cell invasion was determined using CyQUANT NF dye (Invitrogen), as described in the Materials and Methods. The plot displays the statistical results from three independent experiments. *Bars*, mean ± SD. *p<0.05.

In the wound healing assay, the cells ectopically expressing GZB displayed an attenuated migration effect, which was consistent with our previous report. Notably, the cells expressing GZBDK had no effect on cell migration, whereas the cells expressing GZBDZ had moderately attenuated cell migration (Figure **2A**). A similar phenomenon was observed in a Boyden chamber cell migration assay (Figure **2B**). Moreover, our previous study suggested that ZBRK1 can repress the invasion ability of cancer cells [14], which prompted us to assess the contributions of the N- and C-terminal regions of ZBRK1 to cell invasion. When we assessed the invasion effect using Boyden chambers containing Matrigel, the GZBDK-expressing cells lost the effect (Figure **2C**). This finding suggested that the N-terminal KRAB domain of ZBRK1 is critical for the inhibition of cell migration and invasion.

### KAP1 promotes cell migration and invasion

**Figure 3:**
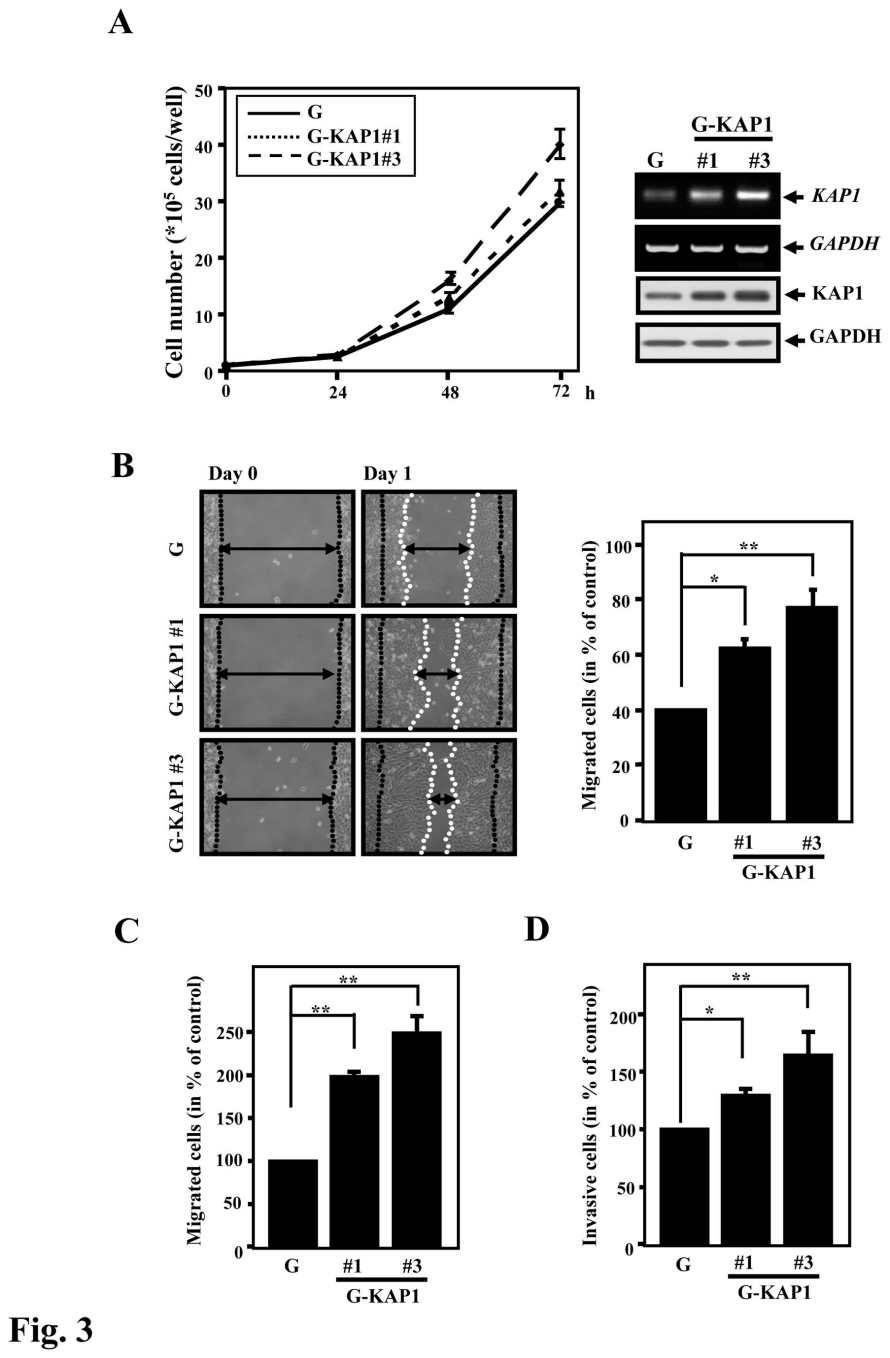
KAP1 enhances cell migration and invasion. **A**, *Right*, HeLa cells were stably transfected with EGFP-KAP1 expression constructs. KAP1 mRNA and protein levels were assessed by reverse transcription-PCR and Western blotting. *Left*, KAP1 expression scarcely induced cell proliferation. Equal numbers of EGFP (G) or EGFP-KAP1 (G-KAP1#1 or G-KAP1#3) HeLa cells were seeded, and the viable cell number was determined at the indicated times. **B**, The wound-healing migration assay was performed with the control and stable KAP1-expressing cells. The cell-free gap (i.e., the “wound”) was created using a culture insert. Representative images of wound sealing were collected on day 0 and the next day (day 1). *Right*, the level of cell migration into the gap quantified as the percentage of wound sealing. *Columns*, the average of three independent measurements; *bars*, mean ± SD. **p<0.01; *p<0.05. **C**, KAP1 enhances the migration of cancer cells. Cells were seeded in a transwell chamber, and the level of cell migration was determined. **D**, KAP1 enhances the invasiveness of cancer cells. Cells were seeded in a BD matrix gel layer, and the level of cell invasion was determined using CyQUANT NF dye, as described in the Materials and Methods. *Columns*, the average of three independent measurements; *bars*, mean ± SD. **p<0.01; *p<0.05.

As shown in Figure 2C, cells expressing ZBRK1 with a deletion of the KRAB domain lose the ability to suppress cell proliferation, migration and invasion. This finding suggested that KAP1 may be important for the proliferation, migration and invasion of cancer cells. To test this hypothesis, we generated HeLa cells ectopically expressing KAP1 (G-KAP1) (Figure **3A** **, right panel**) and performed cell proliferation, wound healing and cell migration/invasion assays. As demonstrated by the cell proliferation assay, the HeLa cells ectopically expressing KAP1 showed a slight increase in their growth rate in a dose-dependent manner (Figure **3A** **, left panel; compare KAP1 expression between clones 1 and 3**). *In vitro* cell migration and invasion assays demonstrated that cells ectopically expressing KAP1 have a more expedient healing effect and higher invasion activity than the control cell line (Figure **3B–D**). These results suggested that KAP1 can promote cancer cell migration and invasion.

### ZBRK1 represses KAP1 transcription through suppressing its promoter activity

**Figure 4:**
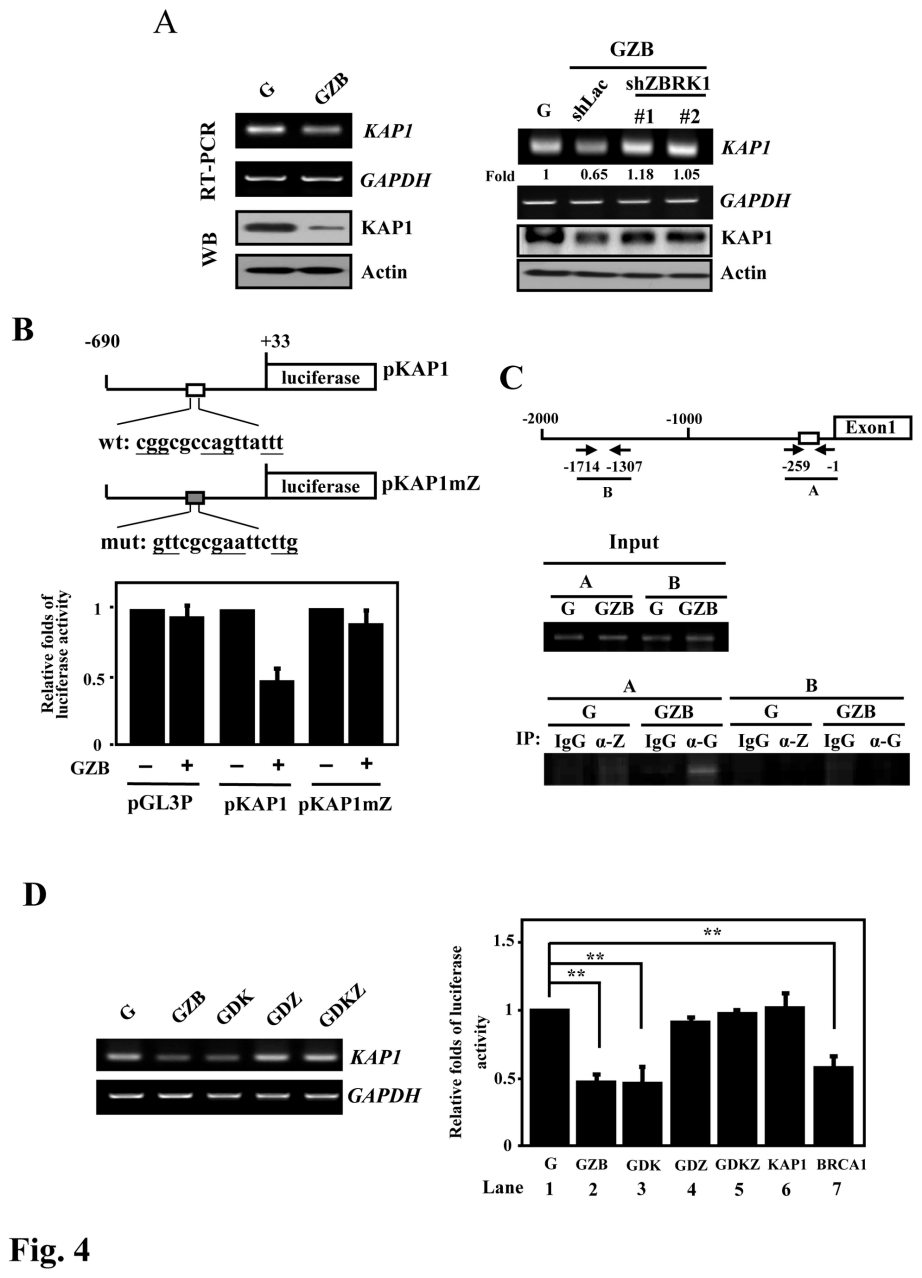
ZBRK1 represses KAP1 promoter activity. **A**, *Left*, ZBRK1 inhibits KAP1 transcripts in HeLa cells. The total RNA and lysates of EGFP (G) and EGFP-ZBRK1 (GZB) HeLa cells were harvested for RT-PCR and Western blot analyses. *Right*, the loss-of-function ZBRK1 enhances KAP1 transcripts. Stable ZBRK1-expressing cells were incubated with lentiviral shRNA against ZBRK1 or the control. The total RNA of infected cells was analyzed using RT-PCR. Human GAPDH served as a control. **B**, ZBRK1 inhibits the KAP1 reporter. *Top*, schematic representation of the luciferase reporter constructs containing the KAP1 promoter with wild-type or mutant ZBRK1- binding motifs. The sequences of the wild type (wt) and mutant (mut) ZBRK1-binding motifs are shown. HeLa cells were co-transfected EGFP-ZBRK1 (GZB) with pGL3 promoter reporter (pGL3p), wild-type KAP1 or mutant ZBRK1 binding motif KAP1 reporters. Lysates of the transfectants were harvested after 12 h for the luciferase assay. The relative fold change in luciferase activity of the various KAP1 reporter constructs are shown after normalization to the wild-type KAP1 reporter. *Bars*, mean ± SD. **C**, ZBRK1 binds to the KAP1 promoter *in vivo*. The sheared formaldehyde cross-linked chromatins, extracted from HeLa cells stably expressing EGFP (G) or EGFP-ZBRK1 (GZB), were immunoprecipitated with the indicated antibodies: control IgG (gG), ZBRK1 (-Z) and GFP (-G). PCR amplification with specific primers targeting the *KAP1* promoter was performed, as described in the Materials and Methods. The figure represents the PCR products obtained using primers specific for the KAP1 promoter region, as shown in the top panel. **D**, *Left*, the C-terminally deleted ZBRK1 mutant reverses the suppressive effect on KAP1 transcripts. The expression levels of KAP1 in HeLa cells exogenously expressing EGFP (G), EGFP-ZBRK1 (GZB), EGFP-ZBRK1 with KRAB domain deletion (GDK), EGFP-ZBRK1 with CTRD domain deletion (GDZ) or EGFP-ZBRK1 with both KRAB and CTRD deletion (GDKZ). A RT-PCR assay was conducted with specific primers of indicated genes. *Right*, BRCA1 is important for the ZBRK1-mediated inhibition of KAP1 reporter activity. HeLa cells were co-transfected with the KAP1 reporter and EGFP (G), GZB, GDK, GDZ, GDKZ, KAP1 or BRCA1 expression vectors. Lysates of the transfectants were harvested after 12 h of transfection for luciferase assay. The data shown are the mean ± SD.

Several reports suggested that ZBRK1 negatively regulates the transcription of the *GADD45*, *ANG1* and *p21* genes [3,15,17]. Interestingly, we observed that KAP1 expression was attenuated in cells expressing ectopic ZBRK1 (Figure **4A** **, left panel**). As further confirmation, the attenuation of ZBRK1 resulted in an increase in steady-state KAP1 mRNA and protein levels (Figure **4A** **, right panel**). To assess whether the change was due to the inhibition of KAP1 promoter activity or posttranscriptional regulation, we examined the KAP1 mRNA turnover rate after treatment with actinomycin D, an inhibitor of transcription. The measured half-life of KAP1 mRNA was approximately 2 h, and there was no clear difference observed between the parental line and the cells expressing ectopic ZBRK1 (Figure **S2**). The result indicated that the reduction in KAP1 mRNA in response to the ectopic expression of ZBRK1 was likely due to the repression of KAP1 promoter activity, but not through posttranscriptional regulation.

To assess whether ZBRK1 suppresses KAP1 expression through regulation of the promoter region of the KAP1 gene, the promoter region, from -690 to +33, of the KAP1 gene was cloned into the pGL-2 basic vector for reporter assays. The result of the reporter assays demonstrated that ZBRK1 can repress KAP1 reporter activity (Figure **4B**). Importantly, the overexpression of ZBRK1 could not repress the luciferase activity of the KAP1 reporter when the putative ZBRK1-binding motif was mutated (Figure **4B**). We next conducted an *in vivo* DNA binding assay to assess whether ZBRK1 can directly bind to the KAP1 promoter. A ChIP assay was conducted using samples of cross-link extracts from stable EGFP (G) and EGFP-ZBRK1 (GZB) HeLa cells to detect the binding of endogenous ZBRK1 and ectopically expressed EGFP-ZBRK1 to the promoter region of the KAP1 gene. The result of the ChIP-PCR assay showed that ZBRK1 can directly bind to the region containing the putative ZBRK1-binding motif on the KAP1 promoter (Figure **4C**). Taken together, these results suggested that ZBRK1 can repress KAP1 transcription via direct binding to the KAP1 promoter.

ZBRK1 can interact with KAP1 and BRCA1 through the KRAB and CTRD domains, respectively, and serves as a transcriptional repressor. We assessed the contributions of KAP1 and BRCA1 to ZBRK1-mediated KAP1 gene repression. The result of an RT-PCR assay showed that the KAP1 transcript levels were attenuated in the ZBRK1 (GZB) and KRAB-truncated ZBRK1 (GDK) transfectants but not in the CTRD-truncated ZBRK1 (GDZ) or the both KRAB- and CTRD-truncated ZBRK1 (GDKZ) transfectants (Figure **4D** **, left panel, and** Figure **S3**). In addition, a reporter assay was conducted to further assess the potent involvement of KAP1 and BRCA1 in ZBRK1-mediated repression of KAP1 reporter activity. In agreement with the RT-PCR results, the loss of the repressive effect was observed in the GDZ and GDZK transfectants (Figure **4D** **, right panel; compare lanes 2 and 3 with lanes 1, 4 and 5**). Moreover, the ectopic expression of BRCA1 but not KAP1 repressed the activity of the KAP1 reporter (Figure **4D** **, right panel; compare lanes 6 and 7**). These results suggested that KAP1 expression inversely correlates with ZBRK1 levels and that the interaction between ZBRK1 and BRCA1 is essential for ZBRK1-mediated repression of the KAP1 reporter.

### KAP1 levels are inversely correlated with ZBRK1 levels in cervical cancer specimens

**Figure 5:**
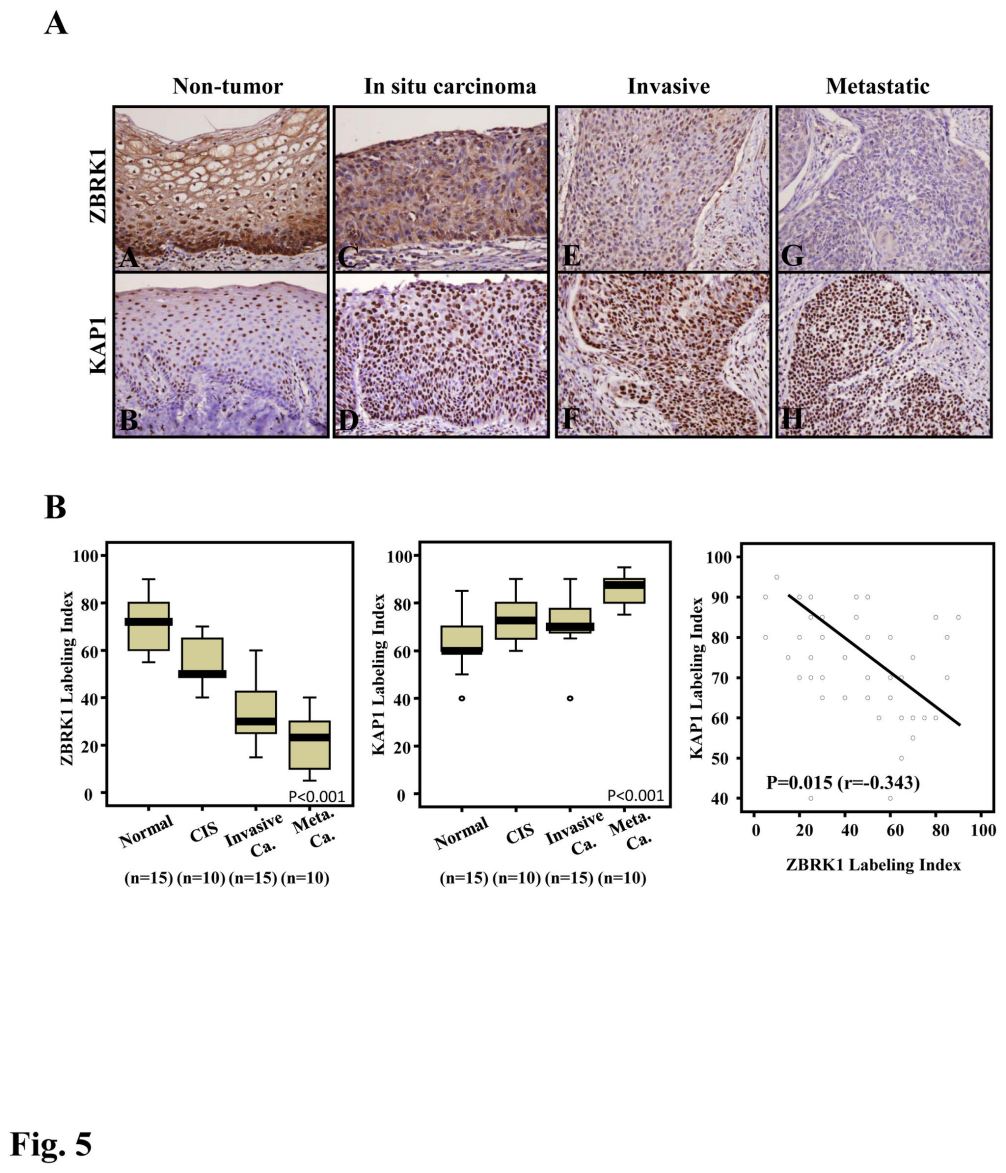
The reduced expression of ZBRK1 is inversely correlated with the increased expression of KAP1 in cervical cancer specimens. **A**, All tumor specimens from 50 patients with different stages of cervical cancer were obtained from surgically resected tissues. ZBRK1 and KAP1 expression was detected in non-tumor epithelium (A, B), in situ carcinoma (C, D) and invasive (E, F) and nodal metastatic carcinoma (G, H) using immunohistochemistry. **B**, A representative image of ZBRK1 demonstrates a significant stepwise decrease, whereas KAP1 had significantly elevated expression. The expression levels of ZBRK1 or KAP1 in normal, CIS, invasive cancer and metastatic cancer were calculated using analysis of variance (ANOVA); p< 0.001 was considered to be significant. ZBRK1 and KAP1 expression levels between the tumor stages were compared using the Chi-squared test, for which two-sided tests of significance were used. A value of p< 0.05 was considered to be significant.

Our previous study demonstrated that ZBRK1 was reduced in cervical cancer specimens [14]. However, the relative levels of ZBRK1 and KAP1 in clinical specimens were unclear. Here, we showed that the endogenous level of ZBRK1 is high in normal cervical tissue and decreases as the tumor progresses, particularly in highly invasive and metastatic cervical cancer specimens (Figure **5A and 5B** **, left panel**). Conversely, the level of KAP1 was low in normal specimens but higher in highly invasive and metastatic cervical cancer specimens (Figure **5A** **, right panel**). Compared with the expression levels of ZBRK1 and KAP1 in the same specimens, an increased expression of KAP1 was correlated with a reduced expression of ZBRK1 (p=0.015).

### ZBRK1 attenuates KAP1-induced invasion and metastasis

**Figure 6:**
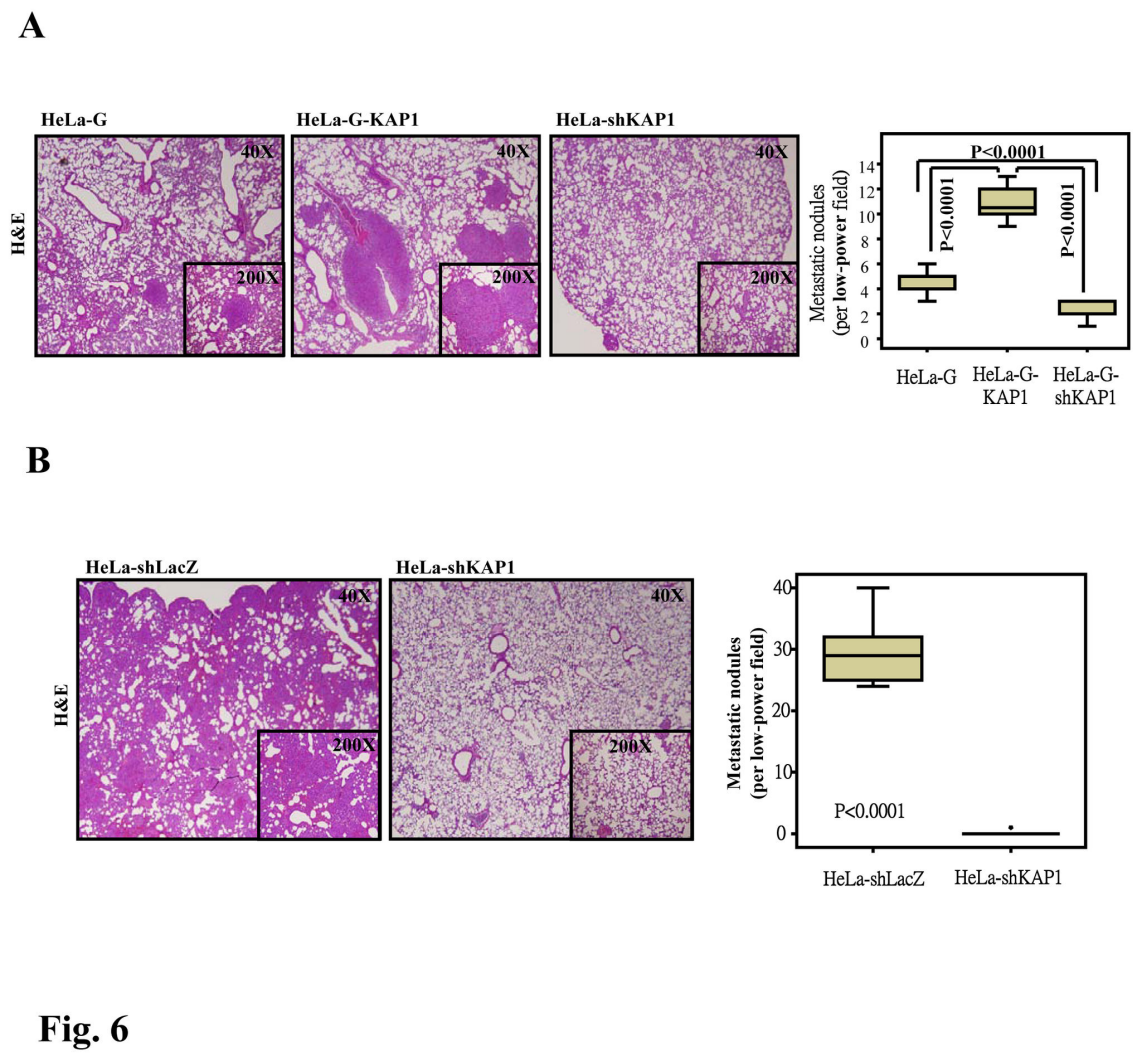
KAP1 enhances cancer cell metastasis *in vivo*. Hematoxylin and eosin (H and E) staining of HeLa cell-derived lung metastasis after 4-6 weeks is shown. Six NOD-SCID mice were tail-vein (A) or subcutaneously (B) injected with parental, KAP1-expressing (G-KAP1) or lentiviral shRNA against KAP1 (shKAP1)-expressing HeLa cells. Representative images of H&E staining sections from metastatic tumor lung tissues are displayed. Magnification: 40 X and 200 X. Analysis of both groups indicated that KAP1 expression significantly affects the metastatic potential of HeLa cells.

In Figures 3 and 4, we demonstrate that a loss of ZBRK1 enhances KAP1 expression and the ability of KAP1 to promote cancer cell migration and *in vitro* invasion and metastasis. These discoveries motivated us to further confirm whether ZBRK1 can attenuate KAP1-induced invasion and metastasis *in vivo*. Histological analyses illustrated that the number of micrometastatic lesions was markedly reduced in the lungs of mice injected with KAP1-depleted HeLa cells and increased in the lungs of mice injected with HeLa cells that ectopically expressed KAP1 (Figure **6A and 6B**). The results suggested that KAP1 plays a functional role in promoting cell metastasis and invasion. These data further support the suggestion that the loss of ZBRK1-triggered increased KAP1 expression contributes to the metastasis and invasion of cervical cancer.

## Discussion

It has been well documented that ZBRK1 exhibits suppressor activity for cancer metastasis; however, the involvements and contributions of ZBRK1-interacting proteins in this intricate process remains elusive. Specifically, the N-terminal KRAB domain and C-terminal BRCT domain of ZBRK1 are responsible for KAP1 and BRCA1 interactions, respectively. In this study, we demonstrated that the KRAB domain of ZBRK1 predominantly governs cancer cell migration; while the ZBRK1’s ability to suppress cell growth appears to require interactions with both KRAB and CTRD domains. Interestingly, exogenous expression of KAP1 exhibited a marginal effect on cell proliferation (Figure **3A**), implying that KAP1 may not be the only protein that interacts with the N-terminus of ZBRK1. Due to KAP1’s feeble involvement in controlling cellular proliferation, BRCA1 has been speculated to be the one responsible for regulating cancer cell proliferation through its interaction with ZBRK1 [18,19], a conjecture that needs to be further investigated.

Our previous work has identified a number of potential downstream genes of ZBRK1 [14], yet the coordinated and separate effects of BRCA1 and KAP1 on ZBRK1-mediated gene regulation remain unclear. For this, mRNA global analyses were conducted using total RNA harvested from GZB, GZBDK and GZBDZ stable HeLa cell lines. Based on the profiling results, in addition to KAP1, 21 responsive genes were identified and categorized by their responsiveness to N-terminal KRAB domain (APP, CDH2, S100A6 and CLDN3 genes), C-terminal CTRD domain (ODC1, VIM and NFE2L2 genes) and both N- and C-termini (MMP9, SH3GLB2 and MAPK4K genes) (Table **S1**). RT-PCR validation (Figure **S3**) supported that KAP1 and BRCA1 can independently or corporately participate to ZBRK1-mediated gene regulations in cells.

In primary cervical cancer specimens, KAP1 levels are inversely correlated with ZBRK1 levels throughout different tumor stages. Briefly, the expression of ZBRK1 was significantly down-regulated in highly invasive and metastatic cervical cancer compared with non-metastatic cervical cancer. Conversely, the level of KAP1 was low in normal and increases as the cancer progresses (Figure **5**). We know that this change in transcriptional levels can result from either promoter activity and/or posttranscriptional modifications/degradation. We determined that ZBRK1 could directly bind to the KAP1’s promoter to repress its expression. Moreover, the half-life of KAP1’s mRNA did not differ significant from that of cells expressing ectopic ZBRK1 (Figure **S2**). Together, these results indicated that KAP1 gene was a downstream target of ZBRK1. We also discovered that KAP1 mRNA expression is reduced in cells ectopically expressing ZBRK1 and GZBDK but not GZBDZ. It was consistent our observation that KAP1 reporter activity was low in cells ectopically expressing BRCA1 but not KAP1. These finding strongly suggested that ZBRK1’s inhibition of KAP1 transcription was critically regulated by the ZBRK1/BRCA1 interaction.

KAP1 is a well-studied transcriptional repressor that binds to KRAB domains of zinc finger proteins [20]. We here show that KAP1 can interact with ZBRK1 to promote cancer cell migration by *in vitro* and clinical analyses. Moreover, we demonstrated that ZBRK1 assumes a repressive role in attenuating KAP1-induced cancer invasion. These results prompted us to explore if the manifestation of KAP1-enhanced mobility is due to the loss of KAP1 promoter repression by ZBRK1 or the lack of ZBRK1/KAP1 protein–protein interaction. Ectopically expression of ZBRK1 in CMV promoter-driven KAP1 expressing cell did not alter the ability of KAP1 to promote cell migration and invasion (Figure **S4**), suggesting that the protein–protein interaction between ZBRK1 and KAP1 has a minimal effect on the KAP1-mediated cancer cell migration.

There have been a number of indirect evidences seen in recent studies that suggest KAP1’s role to promote tumorigenesis in addition to cancer cell migration. First, a proximal transcriptional factor (CBF-A) forms a complex with KAP1 and FTS-1 that activates transcriptional regulators of epithelial-mesenchymal transition. Moreover, there is evidence indicating that the RNA recognition motif (RRM) in CBF-A may interact with the PHD domain of KAP1 [21]. Secondly, the interaction of CBF-A to KAP1 bound to the FTS-1 promoter element may be a proximal activator of EMT [13]. These results suggest that KAP1 also plays a role in metastasis. Thirdly, KAP1’s interaction with MDM2 contributes to p53 functional regulation [22], suggesting KAP1 also plays a role in cancer by inhibiting p53. These findings raised two important issues: What is ZBRK1’s role in these intricate regulations and how is KAP1 regulated by ZBRK1 and BRCA1? The lab is currently actively pursuing the answers to these questions.

There have been some preliminary studies that investigate how epigenetic determinants can reprogram the expression of genes with regards to cancer metastasis. For example, the polycomb group protein EZH2 catalyses histone modifications, promotes DNA methylation, and is a predictive marker of invasive growth in metastatic prostate and breast cancers [23,24]. Also, the activation of the β-catenin-reptin chromatin-remodeling complex leads to silencing of the metastasis suppressor gene *KAI1* [25]. KAP1 was previously identified as a molecular scaffold to regulate chromatin structure through its interaction with Mi-2a, a component of the NuRD histone deacetylase complex [7]; methyltransferase SETDB1 [8]; and heterochromatin protein1 (HP1) family [9,10], making KAP1 the first potential epigenetic regular that promotes cell metastasis. This discovery warrants the identification of the components in the KAP1-mediated complex that participates in the promotion of metastasis.

In this work, we demonstrated that KAP1 has the ability to promote cancer cell metastasis without affecting cancer cell proliferation. Moreover, KAP1 can promote the invasion and metastasis of cancer cells. We also uncovered a novel function of ZBRK1 as a transcriptional repressor of the KAP1 gene. Of clinical relevance, we observed that the expression levels of ZBRK1 were significantly decreased, while the levels of KAP1 were significantly increased in aggressive cervical cancer samples. Taken together, our results indicate that ZBRK1 serves as a suppressor of cancer metastasis by modulating metastasis-related genes via the transcriptional repression of KAP1.

## Materials and Methods

### Patients and tumor specimens

The institutional review board of Chi Mei Medical Center (Tainan, TAIWAN) had approved the study by using formalin-fixed tissue of cervical cancer for this study (IRB100-11-009). Available paraffin-embedded tissue blocks were retrieved from 10 in-situ carcinomas (CIS) [26], 15 invasive carcinomas, and nodal metastases of another 10 invasive carcinomas, as well as 10 non-tumor cervical epithelial samples. The institutional review board approved us to obtain samples from our (Chi-Mei Medical Center) Biobank. As a rule, all samples should be enrolled only with patient agreements and with completed informed consent. Since the samples are then disconnected with their identifiable personal information, further informed consent cannot be available and is not required.

### Cell Culture and Stable Cell Lines Establishment

Cervical cancer cell lines were maintained in complete medium containing DMEM, 5% fetal bovine serum (FBS), 100 g/mL of streptomycin, and 100 units/mL of penicillin. HeLa cells stably expressing enhanced green fluorescent protein (EGFP), EGFP-ZBRK1 or EGFP-KAP1 were generated using either hygromycin B or G418 selection.

### Assay for cell proliferation and focus formation

For the cell proliferation assay, daily cell culture samples were counted after trypsinization using a Neubauer counting chamber. Viability was assessed by trypan blue exclusion. For the focus-forming assay, HeLa cells stably expressing EGFP, EGFP/ZBRK1, EGFP/ZBRK1 deleting KRAB domain, EGFP/ZBRK1 deleting Zn finger domain or EGFP/KAP1 were plated at low density (300 cells per plate) in 100-mm dishes cultured with DMEM containing 5% FBS. The medium was changed every 3 ± 4 d. After 2 wk, the number of colonies was counted using the Sigmascan software program by staining cells with 2% methylene blue in PBS. All of the above experiments were performed in triplicate.

### Reverse transcription-PCR

Total RNA was extracted using TRIzol RNA extraction agent (Invitrogen). The isolated RNA was subjected to a reverse transcription reaction using SuperScript III (Invitrogen) for cDNA production. A PCR was performed using the following specific primer pairs: KAP1, 5’-GACCTGAAGGAGGAGGATGG-3’ and 5’-GGTTCGTGACAGAATAGGGC-3’; GAPDH, 5’-CCATCACCATCTTCCAGGAG-3’ and 5’-CCTGCTTCACCACCTTCTTG-3’.

### Wound healing assay

Before the HeLa cells reached confluence in 6-mm culture plates, a cell-free gap (i.e., the “wound”) was created using a silicon Culture-Insert (ibidi GmbH) placed on the Petri dishes. After removing the silicon insert from the surface, a clean gap was created. Cell migration into the clean region was recorded using a Nikon microscopy system (Nikon Instrument) at 0 and 24 h, and the number of cells in the gap was quantified as the percentage of wound healing.

### Cell migration and invasion assays

Cells were seeded at a density of 5 × 10^4^ cells/well for the migration assay and 1 × 10^5^ cells/well for the invasion assay. The assays were performed in serum-free medium in 24-well (8 µm pore size) BD Invasion Chambers containing MatrigelTM Basement Membrane Matrix (BD Biosciences) and FalconTM migration inserts (8 µm pore size). The inserts were placed into Falcon tissue culture plates containing 5% FBS and incubated at 37°C and 5% CO_2_ for 18 to 24 h to allow for cell migration and invasion through the membrane. The cells that had migrated or invaded to the underside of the insert membrane were dissociated from the membrane. The fluorescence intensity of these cells was detected using CyQUANT NF dye (Invitrogen) and a Fluoroskan Ascent Microplate Fluorometer (Thermo) with excitation at 485 nm and emission detection at 520 nm. Cell migration and invasion activity was calculated as the percentage of fluorescence relative to the controls.

### Experimental metastasis and spontaneous metastasis assays

Female, 4- to 6-week-old nonobese diabetic-severe combined immunodeficient (NOD-SCID) mice were obtained from the National Laboratory Animal Center and fed with Laboratory Autoclavable Rodent Diet (LabDiet). All the mice experiments in study are approved and followed by animal use protocol in NCKU (IACUC approval No. 97051). Six mice were injected with 1 × 10^6^ HeLa cells expressing EGFP, EGFP/KAP1 or lentiviral shRNA against KAP1 (shKAP1). The cells were washed and harvested in PBS and injected i.v. through the lateral tail vein in a volume of 0.1 mL. At 14 d after injection, when the mice had not died but experienced significant morbidity, all of the mice were sacrificed, and their lungs were removed. The number of metastatic nodules was determined by analyzing the entire H&E-stained serial lung sections using light microscopy in low-power fields (40 X) and then confirming in high-power fields (200 X). In the spontaneous metastasis assay, six female, 4- to 6-week-old NOD-SCID mice were injected with 1 × 10^6^ HeLa cells or shKAP1-expressing HeLa cells. The cells were washed and harvested in PBS and injected s.c. into the flank of mice in a volume of 0.1 mL. The tumors were measured with calipers, and volumes were calculated as follows: V = height × width × depth. When the tumors reached a volume of 1000 mm^3^, usually on day 14 after injection, the tumors were removed. Next, 21 days after removal of the primary tumor, the mice were sacrificed, and the extent of metastasis per lung was evaluated by H&E staining.

### Chromatin immunoprecipitation (ChIP)-PCR assay

A ChIP assay was performed as described by Furuta et al. [3], with minor modification. Briefly, sheared chromatin fragments were immunoprecipitated with antibodies specific to GFP (Roche), ZBRK1 (GTX112053) or control mouse IgG at 4°C overnight. After dissociating the DNA from the immunoprecipitated chromatin, the DNA was amplified using PCR. For PCR amplification of specific regions (A and B) of the KAP1 genomic locus, the following sets of primers were used: Fragment A primers: forward, 5’-AGGTTCGCATACCCCACTGGCGGAT-3’ and reverse, 5’-CGCTCGCAGAAAGAGCCGAGGCCGG-3’; Fragment B primers: forward, 5’-TCCTCAGACAGGATTCTCCCCACAC-3’ and reverse, 5’-CCTCCCAAAGTGCTGGAATTACAGG-3’. The PCR profile consisted of 38 cycles of 30 sec at 94°C, 25 seconds at 56°C and 1 minute at 72°C.

### Reporter plasmids and luciferase assay

The reporter plasmids bearing the different fragments of the human KAP1 promoter were generated by PCR using genomic DNA as the template. The primers for the PCR reaction were as follows: KAP1/-690, 5’-GGGGTACCCCTCCTGGGCTAAACGGAGCTTCC-3’ and KAP1/+33, CCCAAGCTTACAAGCACAAGCACAACCGCTC-3’. The PCR fragments were cloned into the multiple cloning sites of the promoter-less vector, pGL2-basic vector, and verified by DNA sequencing. The mutant ZBRK1-binding motifs on the KAP1 promoter were created using a site-directed mutagenesis kit (Stratagene) and then inserted into the pGL2-basic vector. These reporters were introduced into HeLa cells using Lipofectamine 2000 reagent according to the manufacturer’s instructions. The lysates from the transfectants were harvested for use in the luciferase assay. The total amount of DNA for each experiment was matched with the empty backbone vector.

### Lentiviral shRNA

The lentiviral expression vectors included pLKO.1-shLuc, which contained 5’-CTTCGAAATGTCCGTTCGGTT-3’, and pLKO.1-shKAP1, which contained 5’- GACCACCAGTACCAGTTCTTA-3’. These vectors were obtained from the National RNAi Core Facility located at the Genomic Research Center of the Institute of Molecular Biology, Academia Sinica. Virus was produced as described using Lipofectamine 2000 to cotransfect Phoenix cells with the pLKO.1-shLuc or pLKO.1-shKAP1 vector along with pMD2.G and psPAX2.

### Immunohistochemical staining and assessment of ZBRK1 and KAP1 expression

Prior to staining, 3 µm-thick tissue sections were cut onto pre-coated slides from paraffin-embedded tissue blocks, deparaffinized using xylene and rehydrated using ethanol washes. For antigen retrieval, the slides were heated by microwave treatment in a 10 mM citrate buffer (pH 6.0) for 7 min. After blocking endogenous peroxidase with 3% H_2_O_2_, the slides were washed with Tris-buffered saline for 15 min and then incubated with a primary antibody targeting ZBRK1 (GTX112053, 1:100; GeneTex) or KAP1 (A300-274A, 1:50; Bethyl Laboratories) for 1 h. Primary antibodies were detected using the ChemMate DAKO EnVision Kit (K5001; Dako, Carpinteria). The slides were incubated with a secondary antibody (Dako REAL^TM^ Link biotinylated secondary antibody) for 30 minutes, developed with 3, 3-diaminobenzidine for 5 minutes and then counterstained with hematoxylin. Immunoexpression was scored by an expert pathologist (C. F. Li), and the percentage of tumor cells that demonstrated moderate to strong nuclear reactivity was recorded for each specimen.

### Statistical analyses

Statistical analyses were performed using the SPSS 14 software package. The comparisons and correlations between ZBRK1 and KAP1 expression status and the tumor statuses were calculated by Mann–Whitney U test, Kruskal-Wallis test or Spearman’s correlation test as appropriate. The statistics in animal experiments was performed by Mann–Whitney U test. For all tests, two-sided tests of significance with p< 0.05 were considered significant.

## Supporting Information

Figure S1 **Schematic representation of various ZBRK1 expression vectors in this study.** EGFP-ZBRK1 (GZB) and EGFP-ZBRK1 without the KAP1 interaction domain (GZBDK), BRCA1 interaction domain (GZBDZ) and both KAP1 and BRCA1 interaction domains (DZBDKZ).
(TIF)

Figure S2 **ZBRK1 has no effect on KAP1 mRNA stability.** *Left*, EGFP (G) and EGFP-ZBRK1 (GZB) HeLa cells treated with RNA synthesis inhibitor, actinomycin D (5 µg/ml). Total RNA of EGFP (G) and EGFP-ZBRK1 (GZB) HeLa cells were harvested for RT-PCR at indicated times. Expression levels of KAP1 were detected, with GAPDH as a loading control. *Right*, relative folds between KAP1 and GAPDH at G and GZB HeLa cells, respectively.
(TIF)

Figure S3 **Global genes expression profile changes in ZBRK1 and ZBRK1 mutants.** The transcripts of EGFP (G), EGFP-ZBRK1 (GZB), truncated ZBRK1-GZBDK and GZBDZ HeLa cells were harvested and subjected to microarray analysis as described in the Materials and Methods. Gene expression levels were confirmed by RT-PCR using human GAPDH as the loading control.
(TIF)

Figure S4 **Ectopically expressed ZBRK1 has no effect on KAP1-enhanced cell migration and invasion.** EGFP-KAP1-HeLa (G-KAP1) alones #1 and #3 were transfected with ZBRK1 expression vectors. The transfectants were seeded on BD matrix gel layer. The levels of cell migration and invasion of indicated transfectants were analyzed using CyQUANT NF dye (Invitrogen) as described in Materials and Methods. The number of migration and invasion of experimental cells were normalized with EGFP-HeLa cells (G).
(TIF)

Table S1 **List of common ZBRK1 and ZBRK1 deletion construct-mediated gene profiling.** The global profiling was performed on the Phalanx Human whole-genome OneArray^TM^. Over two hundred genes (a fold-change > 1.5 and p < 0.05 was considered significant) responded to the stably expressing ZBRK1 and truncated ZBRK1 (GZBDK and GZBDZ) in HeLa cells.
(TIF)
