# Supplementary figures and images for: Loss of ZBRK1 Contributes to the Increase of KAP1 and Promotes KAP1-Mediated Metastasis and Invasion in Cervical Cancer
Source: PLoS One. 2013 Aug 22;8(8):e73033. doi: 10.1371/journal.pone.0073033 (PMC3749996; doi:10.1371/journal.pone.0073033)

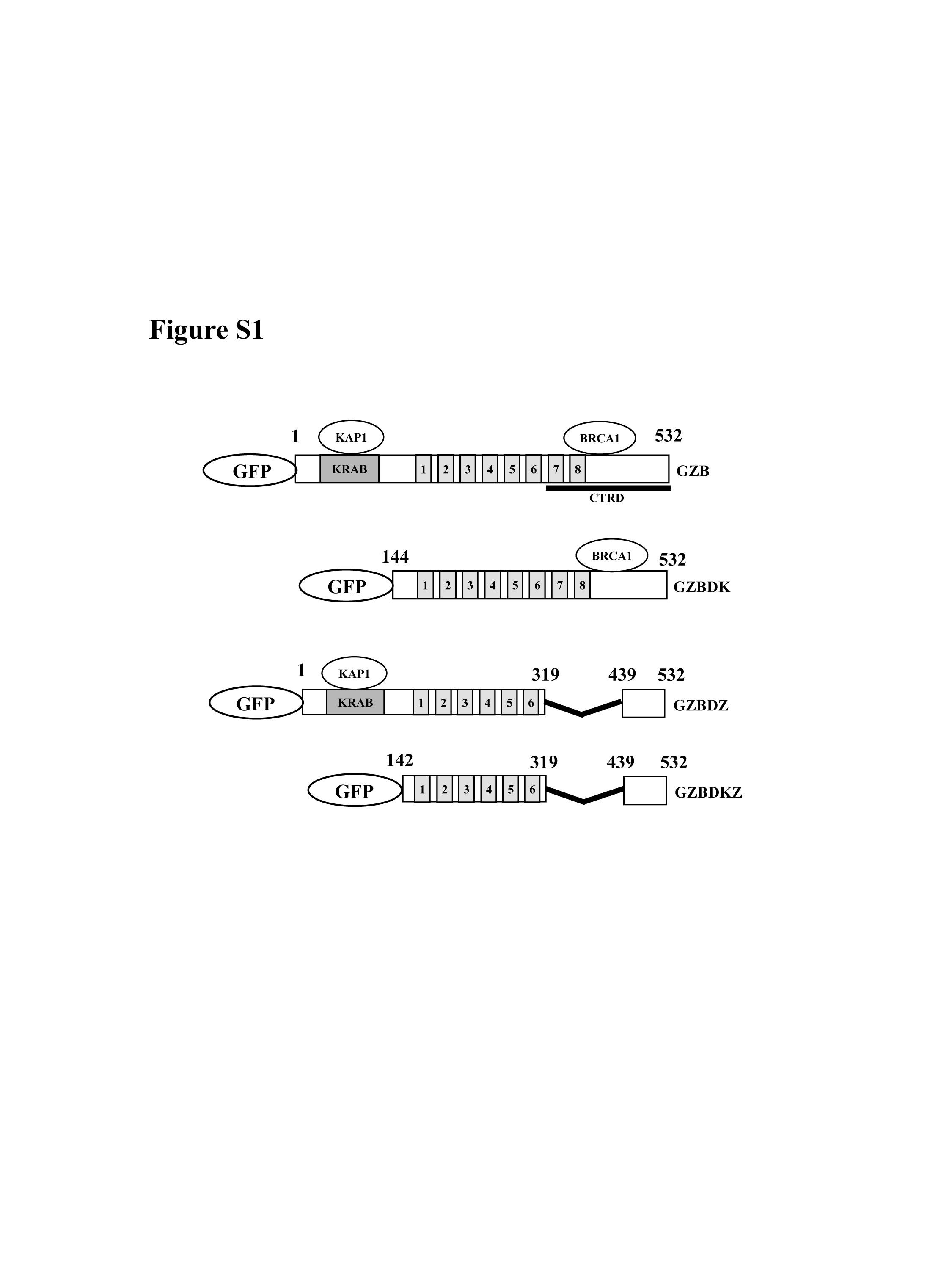

Supplement: Figure S1 — Schematic representation of various ZBRK1 expression vectors in this study. EGFP-ZBRK1 (GZB) and EGFP-ZBRK1 without the KAP1 interaction domain (GZBDK), BRCA1 interaction domain (GZBDZ) and both KAP1 and BRCA1 interaction domains (DZBDKZ). (TIF) [file pone.0073033.s001.tif]

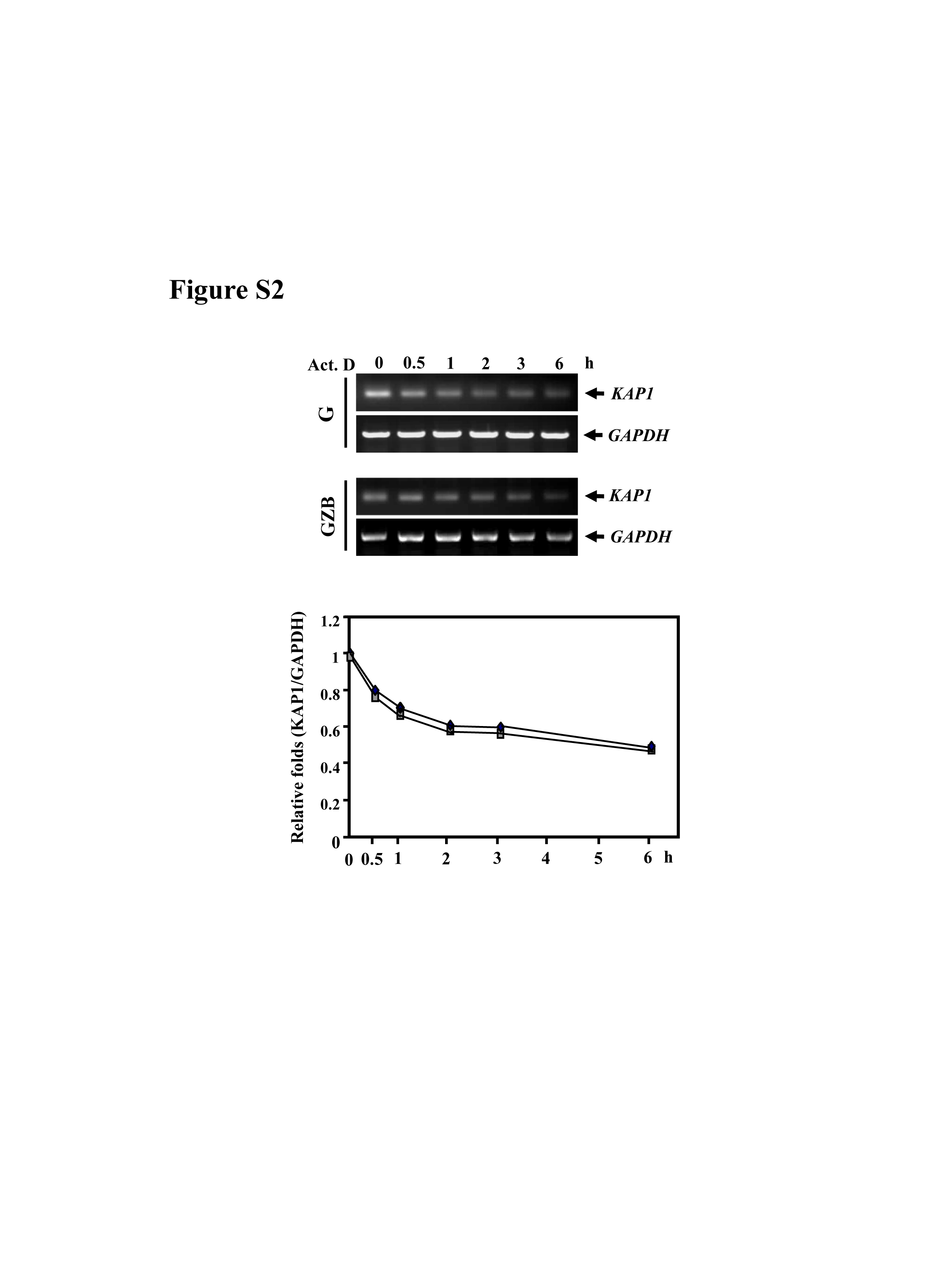

Supplement: Figure S2 — ZBRK1 has no effect on KAP1 mRNA stability. Left, EGFP (G) and EGFP-ZBRK1 (GZB) HeLa cells treated with RNA synthesis inhibitor, actinomycin D (5 µg/ml). Total RNA of EGFP (G) and EGFP-ZBRK1 (GZB) HeLa cells were harvested for RT-PCR at indicated times. Expression levels of KAP1 were detected, with GAPDH as a loading control. Right, relative folds between KAP1 and GAPDH at G and GZB HeLa cells, respectively. (TIF) [file pone.0073033.s002.tif]

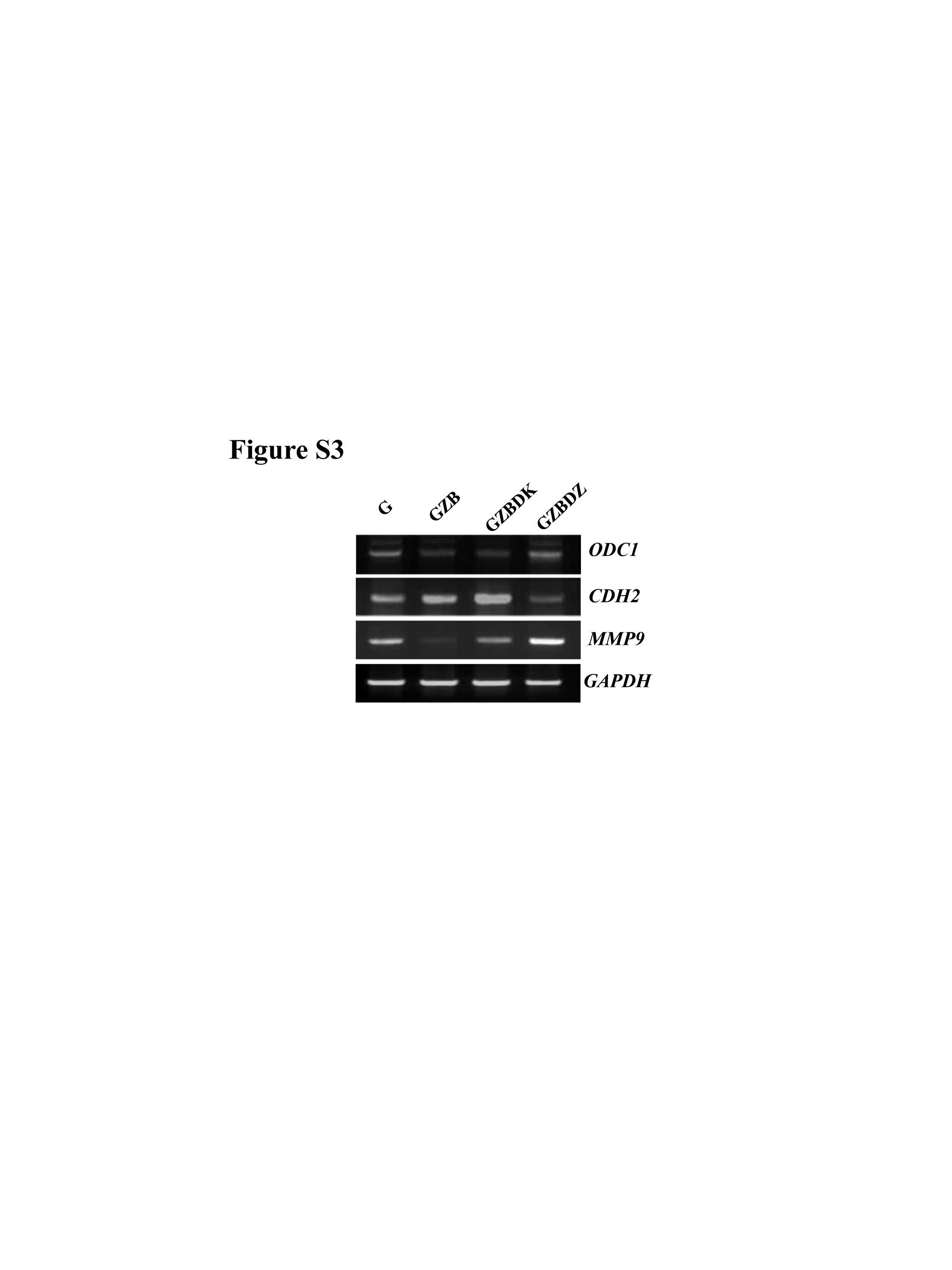

Supplement: Figure S3 — Global genes expression profile changes in ZBRK1 and ZBRK1 mutants. The transcripts of EGFP (G), EGFP-ZBRK1 (GZB), truncated ZBRK1-GZBDK and GZBDZ HeLa cells were harvested and subjected to microarray analysis as described in the Materials and Methods. Gene expression levels were confirmed by RT-PCR using human GAPDH as the loading control. (TIF) [file pone.0073033.s003.tif]

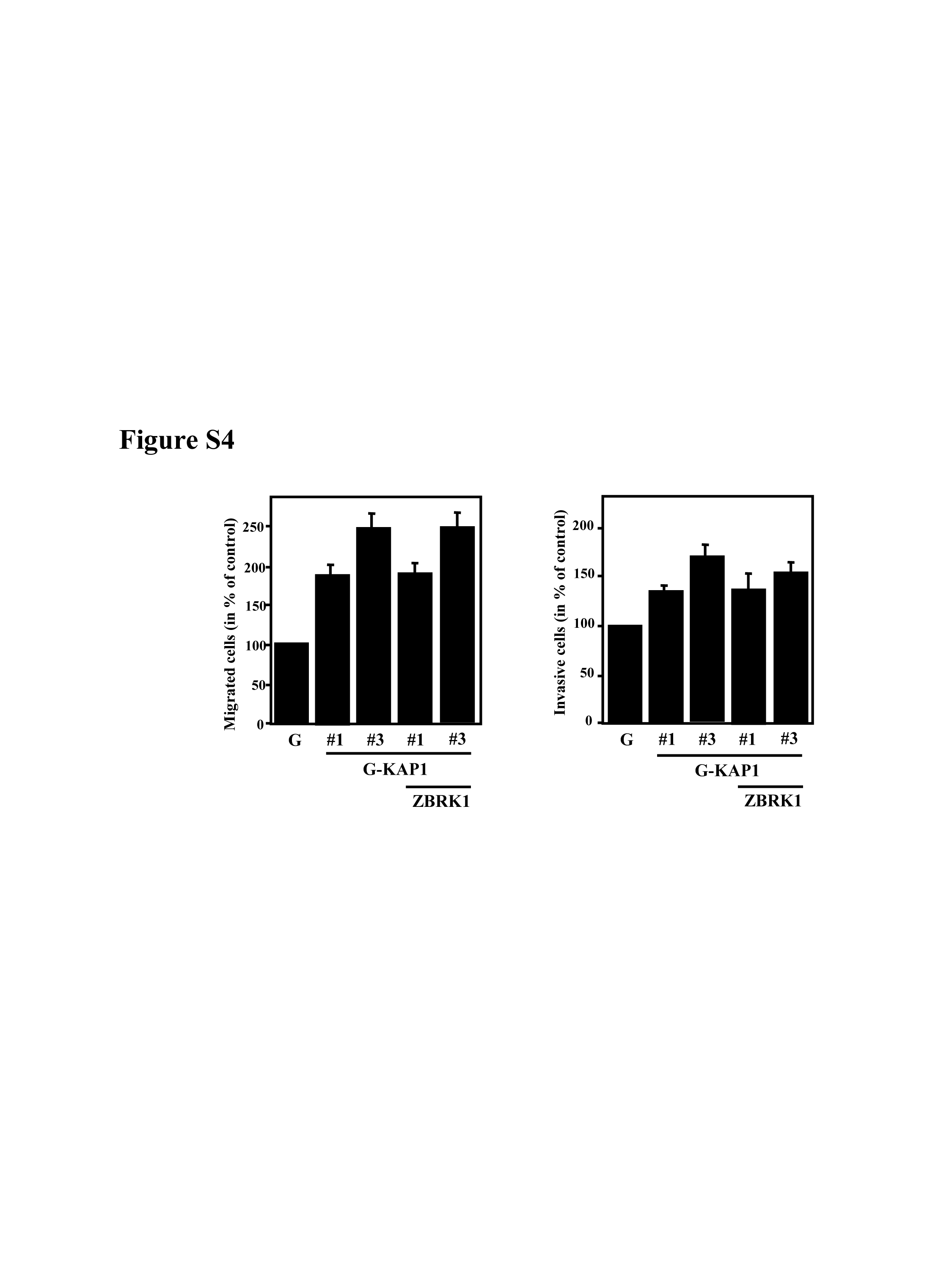

Supplement: Figure S4 — Ectopically expressed ZBRK1 has no effect on KAP1-enhanced cell migration and invasion. EGFP-KAP1-HeLa (G-KAP1) alones #1 and #3 were transfected with ZBRK1 expression vectors. The transfectants were seeded on BD matrix gel layer. The levels of cell migration and invasion of indicated transfectants were analyzed using CyQUANT NF dye (Invitrogen) as described in Materials and Methods. The number of migration and invasion of experimental cells were normalized with EGFP-HeLa cells (G). (TIF) [file pone.0073033.s004.tif]

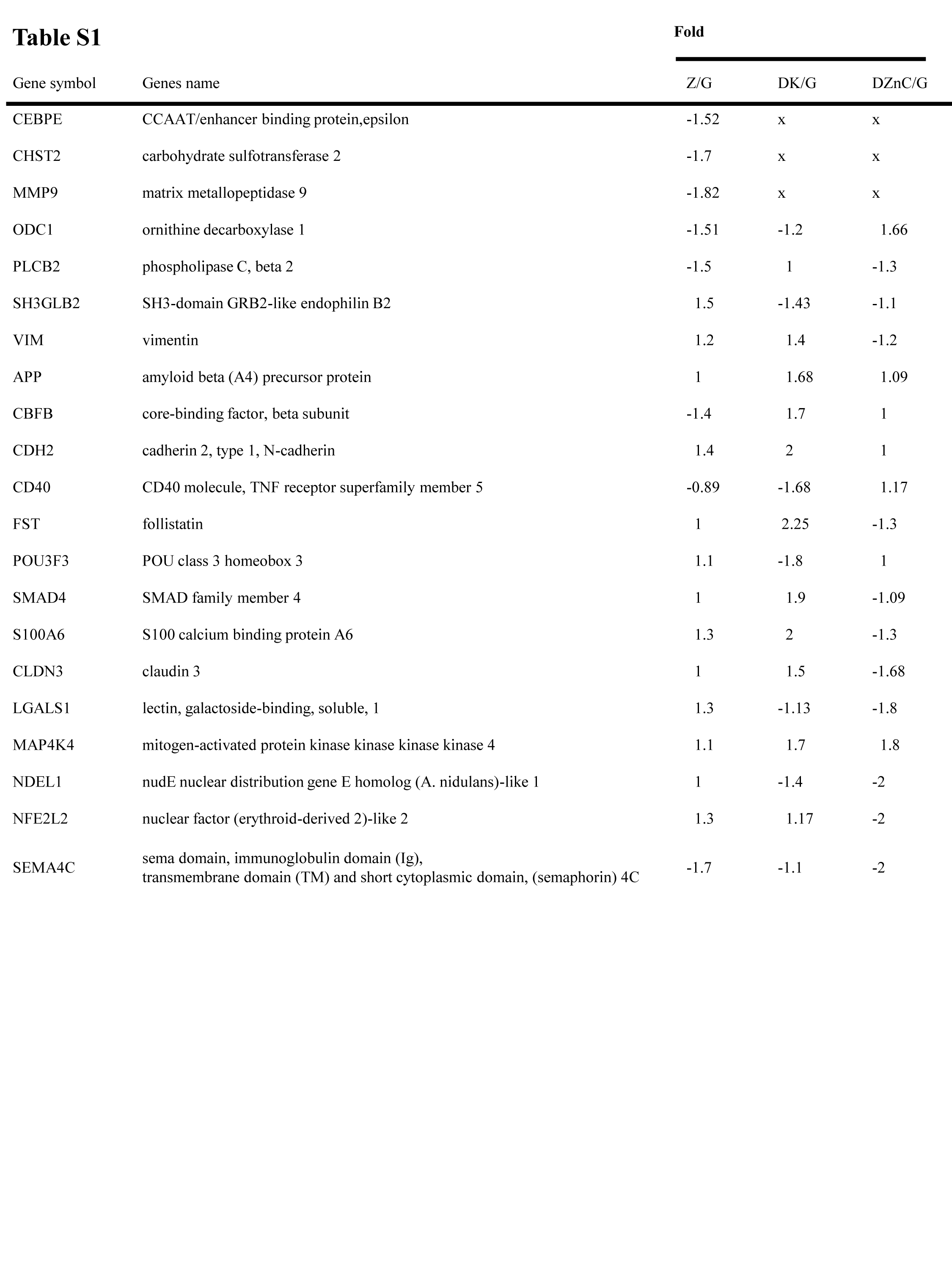

Supplement: Table S1 — List of common ZBRK1 and ZBRK1 deletion construct-mediated gene profiling. The global profiling was performed on the Phalanx Human whole-genome OneArrayTM. Over two hundred genes (a fold-change > 1.5 and p < 0.05 was considered significant) responded to the stably expressing ZBRK1 and truncated ZBRK1 (GZBDK and GZBDZ) in HeLa cells. (TIF) [file pone.0073033.s005.tif]
